# Early distant progression in adult Histone-3 K27-altered diffuse midline gliomas

**DOI:** 10.1007/s11060-025-05238-z

**Published:** 2026-01-13

**Authors:** Niklas Grassl, Abigail K. Suwala, Tobias Kessler, Lukas Bunse, Iris Mildenberger, Michael O. Breckwoldt, Miriam Ratliff, Stefanie Brehmer, Christel Herold-Mende, Nima Etminan, Wolfgang Wick, Felix Sahm, Michael Platten, Katharina Sahm

**Affiliations:** 1 DKTK (German Cancer Consortium) Clinical Cooperation Unit (CCU) Neuroimmunology and Brain Tumor Immunology, German Cancer Research Center (DKFZ), Heidelberg, Germany; 2 Department of Neurology, Medical Faculty Mannheim, MCTN, University of Heidelberg, Theodor-Kutzer-Ufer 1-3, Mannheim, 68167 Germany; 3 DKFZ Hector Cancer Institute at the University Medical Center Mannheim, Mannheim, Germany; 4 Department of Neuropathology, University Hospital Heidelberg, University of Heidelberg, Heidelberg, Germany; 5 DKTK Clinical Cooperation Unit Neuropathology, German Cancer Research Center (DKFZ), Heidelberg, Germany; 6 Department of Neurology, University Hospital Heidelberg, Heidelberg, Germany; 7 National Center for Tumor Diseases (NCT), University Hospital Heidelberg, Heidelberg, Germany; 8 Hertie Network of Excellence in Clinical Neuroscience, Tübingen, Germany; 9 Department of Neuroradiology, University Hospital Heidelberg, University of Heidelberg, Heidelberg, Germany; 10 Department of Neurosurgery, Medical Faculty Mannheim, University of Heidelberg, Mannheim, Germany; 11 Department of Neurosurgery, University Hospital Heidelberg, University of Heidelberg, Heidelberg, Germany; 12 Helmholtz Institute for Translational Oncology (HI-TRON) Mainz, German Cancer Research Center, Mainz, Germany

**Keywords:** Glioma, Histone-3 K27M-Mutation, Diffuse midline glioma, Distant progression, DNA methylation

## Abstract

**Purpose:**

H3 K27-altered diffuse midline glioma (DMG) is a molecularly defined, highly aggressive tumor entity since the 2016 revision of the World Health Organization (WHO) classification of tumors of the central nervous system (CNS). It has been most extensively characterized in children and adolescents and continues to pose significant challenges to treating physicians due to its growth along the midline structures of the brain and its ability to progress distantly leading to poor prognosis. Clinical and molecular determinants of distant progression and its impact on patient survival particularly in adults have not been thoroughly characterized.

**Methods:**

This retrospective multicenter cohort study of 52 adult patients with DMG was analyzed for clinical and molecular predictors of distant tumor progression using gene panel sequencing and genome-wide DNA methylation arrays.

**Results:**

Distant progression including leptomeningeal disease occurred in 10 out of 52 adult patients (19%), seven of which had a primary spinal tumor location (70%, *p* < 0.001) and almost 80% of patients with spinal DMG experienced distant progression. Across the entire cohort, distant progression was associated with worse progression free survival (PFS) (4.5 vs. 13 months; *p* = 0.037). However, we could not identify a genetic or methylation-based signature that was significantly correlated with the occurrence of distant progression.

**Conclusions:**

Adult DMGs with spinal location are at a high risk of early distant progression. As there were no molecular determinants of distant progression from differential methylation, copy number alterations (CNA) or panel sequencing, adult patients with DMG should undergo routine craniospinal MRI, particularly in spinal tumor location.

**Supplementary Information:**

The online version contains supplementary material available at https://doi.org/10.1007/s11060-025-05238-z.

## Key points

• Distant progression occurs in one fifth of adult DMG patients, predominantly in tumors with spinal location.

• Distant progression is associated with shorter progression-free survival and spinal tumor location.

• There are no molecular signatures based on methylation, CNAs or panel sequencing that are associated with the risk of distant progression.

## Introduction

Diffuse midline glioma, H3K27-altered, are highly aggressive central nervous system (CNS) tumors that typically arise in midline structures and are therefore predisposed to disseminate via the cerebrospinal fluid (CSF) compartment. Since their first inclusion in the 2016 World Health Organization (WHO) classification of CNS tumors [1], DMGs have been predominantly characterized in pediatric populations [2], while their clinical and molecular features in adults remain less well defined.

DMGs carry a dismal prognosis independent of tumor location [3, 4], but emerging evidence suggests that DMG represents a heterogeneous tumor entity [5, 6], with age at diagnosis influencing tumor biology and disease progression. In contrast to H3.1-mutant tumors, H3.3 mutations are more frequent in adults [7] and, together with other molecular features such as ATRX loss [8, 9] and FGFR1 mutations [10], are associated with a comparatively better prognosis in adult DMG patients than in children. Stegat et al. identified a H3K27-altered DMG subgroup with an improved prognosis, associated with MAPK mutations and adult age, based on genome-wide methylation data [11]. Age- and localization-dependent differences have also been described in the tumor microenvironment of H3K27-altered DMG [6], which, in addition to stem cell autonomous mechanisms, contribute to the high rate of distant tumor progression compared to other glioma types [12]. Distant progression, defined as the appearance of a new, measurable T2/fluid-attenuated inversion recovery (FLAIR) hyperintense lesion without a continuous connection to the primary tumor, occurs frequently as does leptomeningeal dissemination (LMD) [13, 14] and both contribute to clinical deterioration and treatment failure. A comprehensive study on LMD in 304 patients diagnosed with DMG found that tumors with contrast-enhancing lesions reaching ependyma were associated with LMD [15].

Despite its clinical significance, the impact of distant progression on prognosis, the role of tumor location in determining the risk of distant spread, and whether molecular signatures can predict this phenomenon remain unclear. Moreover, understanding the dynamics of distant progression may inform future clinical trial designs. Ongoing clinical trials testing histone deacetylase inhibitors [16], peptide vaccines [17, 18] and chimeric antigen receptor T cells targeting GD2 [19, 20] do not stratify patients based on their risk of distant progression. Yet many trials, including the clinical trials that lead to the accelerated approval of dordaviprone by the food and drug administration exclude patients with spinal tumor location or leptomeningeal dissemination [21–23]. To date, no established molecular or clinical tools exist to predict which patients are at the highest risk of distant progression.

In this study, we aim to define the clinical and molecular correlates of distant progression in adult DMG by analyzing a retrospective multicenter cohort. Our findings provide new insights into the clinical relevance of distant progression and its implications for patient management.

## Materials and methods

### Study population

In this multicenter, retrospective analysis, we identified 52 patients above 18 years of age with pathologically confirmed H3K27-altered diffuse midline gliomas according to 2021 WHO Classification of Tumors of the Central Nervous System [24]. All but one patient harbored the canonical H3.3 K27M mutation. The remaining case did not show a detectable H3K27M mutation but was confirmed to be H3K27-altered based on immunohistochemistry and genome-wide DNA methylation profiling. Pathological diagnosis was further supported by DNA methylation-based classification [25] with a matching threshold of >0.9 in 36 patients. In the remaining 16 patients H&E staining and H3K27M mutation-specific antibody staining confirmed presence of H3K27M hallmark mutation.

All patients were treated at the University Medical Centers Heidelberg or Mannheim of Heidelberg University between March 2017 and February 2025. Tissue sample collection and data collection and use were done in accordance with local ethics regulations and approval (institutional ethics committee approval ID: 2017–589 N-MA and S-318/2022).

### Clinical outcomes

Progression-free survival defined as time from initial diagnosis on imaging to date of tumor progression was assessed on the basis of imaging follow-up according to Response assessment in neuro-oncology (RANO) criteria [26]. Tumor localization was defined as the largest site of disease or contrast enhancing lesion with greatest diameter in cases where the tumor extended into multiple anatomic sites. Neuro-imaging studies were obtained in twelve-week intervals or earlier in case of clinical signs of progressive disease. Distant tumor progression was defined as the appearance of a new, measurable T2-fluid attenuated inversion recovery (FLAIR) hyperintense lesion without continuous connection to the primary tumor or the occurrence of LMD. Central necrosis was defined as non-enhancing central core that is T1 hypointense, T2/FLAIR hyperintense, shows facilitated diffusion and low relative cerebral blood volume centrally. Overall survival was defined as time from surgery to date of death.

### Methylation analysis

Bioinformatic analysis of Illumina 450k and 850k methylation array data was performed in R version 4.4.1 (2024-06-14) with a customized script using the following packages GEOquery, dplyr, readxl, minfi, minfiData, gplots, dplyr, ggbeeswarm, ComplexHeatmap, epitools, illuminaio, survival, survminer, qqman, IlluminaHumanMethylation450kmanifest, IlluminaHumanMethylationEPICmanifest, IlluminaHumanMethylationEPICanno.ilm10b4.hg19, IlluminaHumanMethylation450kanno.ilmn12.hg19, missMethyl, methylGSA, Rtsne, ggplot2, DMRcate. The Illumina Infinium HumanMethylation450 (450k) and Infinium MethylationEPIC BeadChip (850 K) chip and kits (Illumina, San Diego, CA, USA) were used according to the manufacturer’s instructions.

For the analysis of differentially methylated positions (DMP) loci with single nucleotide polymorphisms were dropped using the function dropLociWithSnps() and differentially methylated regions between patients with distant progression or not including subsetting for tumor location were determined using dmpFinder(). Circos plots showing DMR distribution were constructed with the package “OmicCircos” (version: 1.42.0).

Copy number segments were aggregated to chromosome-arm or whole-chromosome level and annotated as gain or loss for each patient. For distant vs. no distant progression and spinal vs. non-spinal comparisons, the frequency of gains and losses per chromosome was tabulated across patients. Statistical significance was assessed using contingency tables and Fisher’s exact test, with multiple-testing correction (Benjamini–Hochberg).

### Panel sequencing

34 Patients underwent DNA panel sequencing of the genes and fusions listed in supplementary Table 2 with the aim to detect actionable molecular targets. Agilent SureSelect Technology was used to enrich specific target regions. Enriched products were sequenced on Illumina NextSeq at a minimum average coverage of > 1000. Low-quality calls and SNVs with a frequency of ≤ 0.001 in the 1000 genomes database were excluded. CNA analysis was performed on methylation array data using segmentation-based approaches. CNA profiles were not derived from panel sequencing.

### Statistical analyses

Log-rank test and Kaplan-Meier estimates were used to compare survival times between treatment modalities and sexes. p values less than 0.05 were considered significant. Benjamini-Hochberg correction was used to correct for multiple hypothesis testing.

### Data and code availability

Source code and sequencing data can be made available on reasonable request.

## Results

### Patients with distant progression have shorter progression free survival

**Fig. 1:**
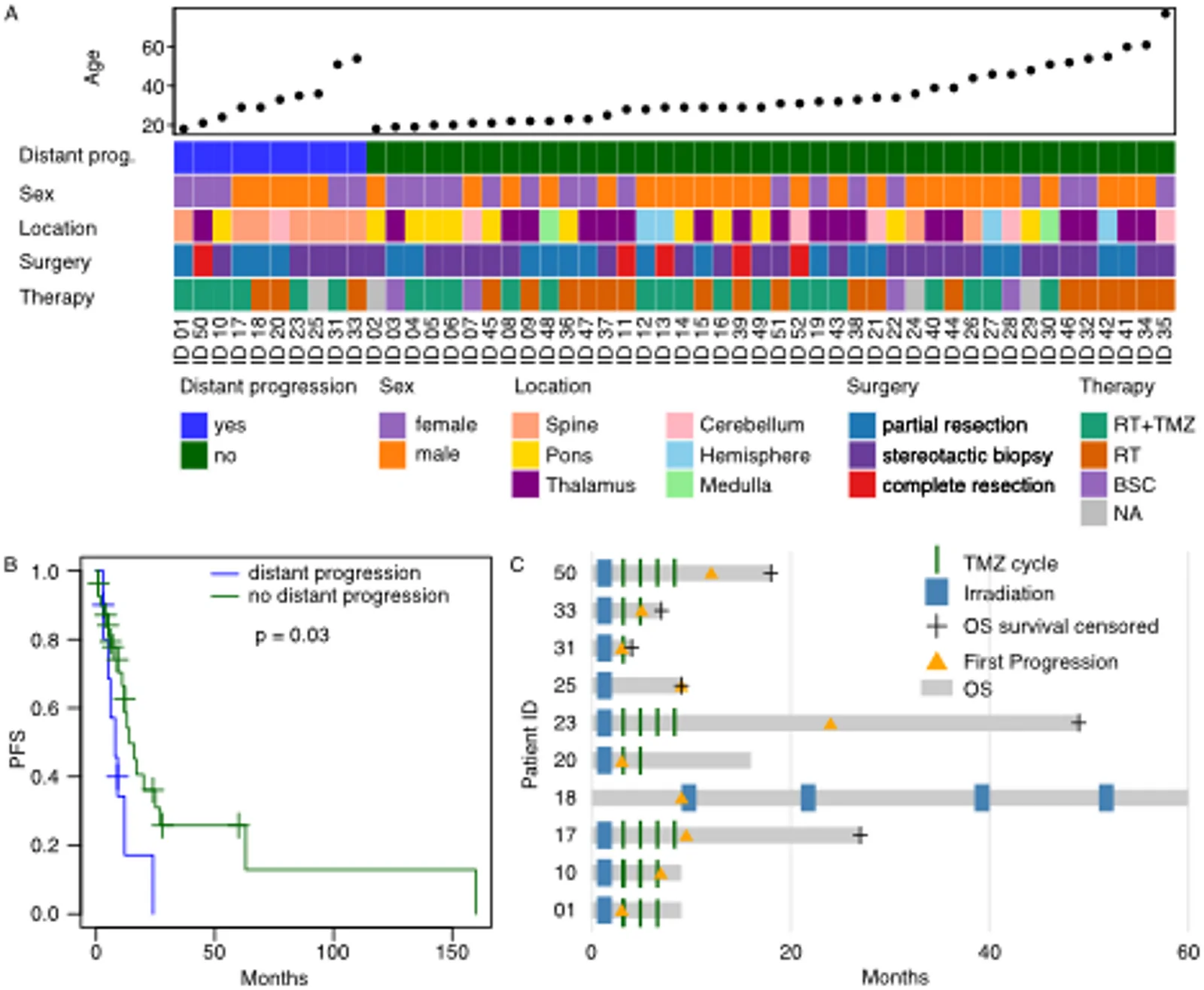
*Distant progression is associated with shorter progression free survival*: (**A**) Treatment characteristics of 52 adult patients with H3K27M altered DMG. Missing values are indicated in grey. RT radiotherapy, TMZ temozolomide. (**B**) Median progression free survival (PFS) was significantly (*p* = 0.03) shorter in patients with distant progression. (**C**) Individual disease trajectories of patients with distant progression. In all 10 cases distant progression was detected on first disease progression indicated with a yellow triangle

In this retrospective multicenter cohort, 10 of 52 adult patients (19.2%) with histologically confirmed H3K27M-mutant DMG experienced distant progression, defined as tumor progression occurring at a non-contiguous site outside the radiation field if intracranial radiotherapy was part of the therapy (Fig. 1A). None of the patients had metastatic disease at initial diagnosis and we did not detect any metastases outside the CNS. The mean patient age at diagnosis was 31.8 ± 13.3 years (mean ± s.d.), with an age range extending up to 77 years. Additional patient characteristics are summarized in Supplementary Table 1.

The median progression-free survival (PFS) from initial diagnosis was 12 months (95%-CI 9–24 months), while the median overall survival (OS) was 41 months (95%-CI 21-NA months) (Supplementary Fig. 1A, B). Neither the extent of resection nor adjuvant treatment with temozolomide significantly affected PFS or OS (*p* = 0.4, *p* = 0.3, *p* = 0.4, *p* = 0.8, Supplementary Fig. 1C-F). Also, we did not observe differences in PFS or OS depending on MGMT promotor status, tumor location or sex (Supplementary Fig. 2A-D).

In all 10 patients, distant progression occurred during first disease progression and patients with distant progression had a significantly shorter median PFS compared to patients without (4.5 vs. 13 months; *p* = 0.037, Fig. 1B, C). This PFS disadvantage did not lead to a difference in median OS (60 vs. 41 months; *p* = 0.85).

### Spinal tumor location as a risk factor for early distant progression

**Fig. 2:**
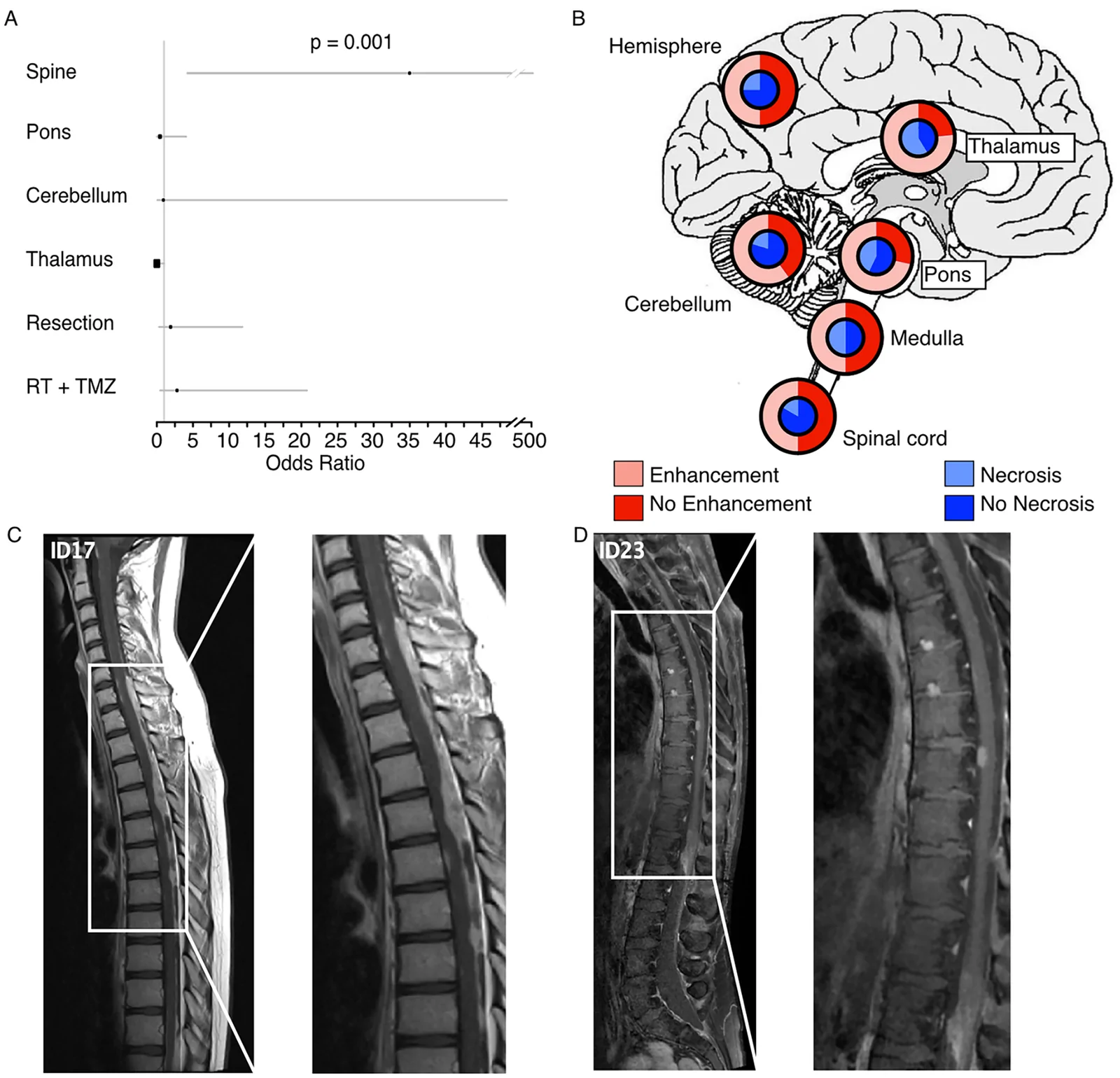
*Distant progression is associated with spinal tumor location* (**A**) Spinal tumor localization was an independent risk factor for the occurrence of distant progression. (**B**) Percentage of samples with contrast enhancement on contrast enhanced T1 MRI sequences at the time of initial diagnosis and of necrosis in subsequent resection or biopsy depending on tumor location. (**C**) Extensive leptomeningeal spinal contrast enhancement in contrast enhanced T1 MRI of a patient with spinal DMG. (**D**) T1 weighted MRI of a spinal DMG with contrast enhancement, but without central necrosis

Notably, distant progression was strongly associated with spinal tumor location (*p* < 0.001, Fig. 2A, Supplementary Fig. 2E): In our cohort, 70% of distantly progressing tumors had a primary spinal location and 7/9 patients with spinal DMG experienced early distant progression. Notably, spinal tumor location alone was not significantly associated with shorter PFS (*p* = 0.1, Supplementary Fig. 2F).

Across tumor sites, 63% of adult DMG showed MRI contrast enhancement and 41% tumor necrosis. In primary spinal DMG, MRI contrast enhancement was detected in 50% of cases whereas central necrosis was less frequent (Fig. 2B, C). In contrast, all distantly progressive tumors showed marked contrast enhancement. Leptomeningeal disease with solid, nodular tumor manifestations along the spinal cord was observed as a common progression pattern of spinal DMG (Fig. 2D).

### Distant progression was not associated with molecular signature

**Fig. 3:**
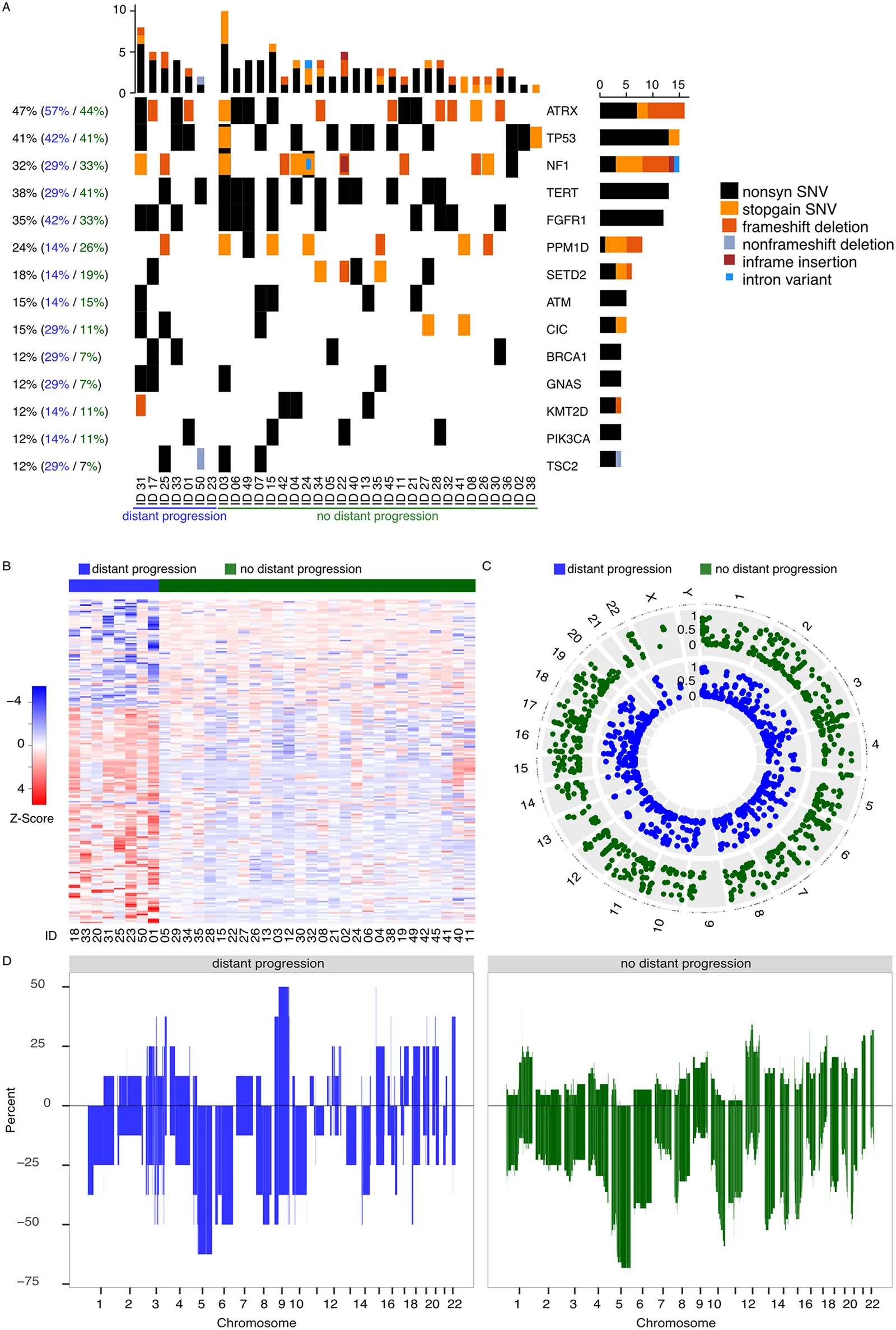
Distant progression occurs more often in spinal tumors: (**A**) Oncoprint of 34 patients that had undergone Panel sequencing for pathophysiologically relevant genes in Supplementary Table 2. Percentages in balck, blue and green indicate frequency of mutations in the respective genes respectively. (**B**) Supervised clustering of differentially methylated CpG sites between cases with distant progression and without distant progression did not yield significantly differentially methylated CpGs after stringent correction for multiple hypothesis testing. Displayed is the Z-scored beta value of all CpGs with unadjusted p-value < 0.005. (**C**) Distribution of CpGs from (**B**) across the genome. The inner circle shows average beta values of cases with distant progression (blue), the outer circle shows average beta values of cases without distant progression (green). (**D**) Percentage of copy number alterations (CNA) in cases with distant progression (blue) and without distant progression (green). No significant differences between CNA profiles were detected

Among the 34 patients who underwent panel sequencing of pathophysiologically relevant genes, seven had a distant progression. The most common mutation apart from H3K27M (*n* = 33, 97%) were in ATRX (*n* = 16, 47%), TP53 (*n* = 14, 41%), TERT promotor (*n* = 13, 38%), FGFR1 (*n* = 12, 35%) and NF1 (*n* = 11, 32%). No significant associations between distant progression and suspected tumor drivers were identified (Fisher’s Exact Test at Benjamini-Hochberg corrected false discovery rate of 5%; Fig. 3A).

Analysis of differentially methylated CpG sites between samples with distant progression or not identified 442 CpG sites with *p* < 0.005 (Fig. 3B). These CpG sites were widely distributed across the genome and showed a slight increase in methylation in tumors with distant progression (Fig. 3C). However, after Benjamini-Hochberg correction for multiple hypothesis testing, no CpG sites or differentially methylated genomic regions remained statistically significant. Restricting the analysis to the 10,000 most variable CpG sites did not alter the results. Also, analysis within anatomical locations did not yield any differentially methylated CpGs between samples with distant progression or not.

Since copy number alterations (CNA) are early events in the evolution of DMG [5], we compared CNA profiles between tumors with and without distant progression, using methylation array data (Fig. 3D). CNAs were highly prevalent across all samples, but no significant differences in CNA patterns were detected between tumors that progressed distantly and those that did not.

## Discussion

In this retrospective cohort study of 52 adult patients with DMG we could not identify molecular predictors of distant progression through analysis of methylation array and panel sequencing of brain tumor-relevant genes. While previous studies have demonstrated that CNAs form early in the evolution of DMGs [5], our findings did not reveal significant differences in the CNA profile between patients with and without distant progression, suggesting that distant progression is not driven by CNAs. However, the statistical power of our study was limited by the relatively small sample size and the necessity to correct for multiple hypothesis testing. These constraints also precluded a meaningful analysis of CNA patterns stratified by tumor location.

CSF analysis, including cytology, was not systematically performed in our cohort, and therefore no conclusions can be drawn regarding the role of early CSF positivity in patients with distant progression. This represents a limitation of our study.

The strongest association with distant tumor progression was observed for spinal tumor location. Spinal tumors are anatomically closer to CSF pathways, which may help explain their higher rate of distant progression. However, our dataset did not allow for a systematic analysis of tumor–CSF proximity, and this question warrants further investigation in future studies.

Notably, cases with distant progression had a shorter PFS but did not exhibit significantly shorter OS compared to patients without distant progression. This discrepancy may be attributed to the high proportion of patients with primary spinal tumors, as spinal tumor progression is likely to become symptomatic earlier, thereby contributing to increased morbidity.

The lack of molecular predictors for distant progression in this cohort may reflect limited statistical power. Furthermore, most publicly available datasets do not systematically report the occurrence of distant tumor progression, hindering the ability to incorporate external datasets and enhance statistical power. Given the infiltrative growth pattern of diffuse gliomas [27], it is plausible that all DMGs disseminate to distant tumor locations microscopically, but only a small minority progress to a size detectable by MRI. Thus, the biological differences between DMGs with distant progression detectable by MRI and those without may be subtle, posing challenges for the identification of robust molecular predictors. In light of this, we recommend that all adult patients with DMG undergo routine craniospinal MRI at diagnosis — analogous to current pediatric recommendations [28] — with particularly attention to cases with spinal tumor location, and again at progression to enable early detection of distant disease. Moreover, while patients with spinal DMG appear to be at particular risk of early distant progression and may therefore benefit from early targeted approaches, the dismal prognosis of DMG overall argues for considering personalized targeted therapy in all patients, if available, in addition to local treatment. Such early intervention may help delay both local and distant progression, thereby minimizing new symptoms and functional decline.

Future studies with larger, multi-institutional datasets that include comprehensive records of distant progression are essential to identify potential molecular predictors and refine patient management. Reliable identification of patients at high risk of metastatic dissemination could eventually justify the use of upfront craniospinal irradiation. In the absence of clear molecular markers, systematic imaging remains the most reliable method for detecting distant progression, particularly in patients with spinal involvement. This approach may improve early detection, facilitate timely therapeutic interventions, and help preserve performance status by mitigating the impact of early distant progression.

## Electronic supplementary material

Below is the link to the electronic supplementary material.

Supplementary Material 1

## Data Availability

Sequencing, clinical, and DNA methylation data generated and/or analyzed during this study are available from the corresponding author upon reasonable request. Due to patient confidentiality and data protection regulations, access to raw clinical data may be subject to institutional review or data sharing agreements. Where applicable, processed datasets and relevant analysis scripts can also be shared upon request.
