# Supplementary material for: Early distant progression in adult Histone-3 K27-altered diffuse midline gliomas
Source: J Neurooncol. 2026 Jan 13;176(2):166. doi: 10.1007/s11060-025-05238-z (PMC12799738; doi:10.1007/s11060-025-05238-z)
Supplement: Supplementary file 1 [file 11060_2025_5238_MOESM1_ESM.docx]

*Supplementary Figure 1: PFS and OS did not differ by treatment:*

(A) Median progression free survival (PFS) was 12 months. (B) Median overall survival (OS) was 41 months. (C-F) Neither PFS (C, E) nor OS (D, F) depended on adjuvant treatment (C, D) nor on mode of resection (E, F). RT radiotherapy, TMZ temozolomide, PR partial resection, CR complete resection, STX stereotactic biopsy.

 *Supplementary Figure 2: PFS and OS did not differ by MGMT promotor methylation or tumor location:*

(A, B) PFS and OS did not differ between the five samples with MGMT promotor methylation (green) and the cases without MGMT promotor methylation (blue). (C-F) Kaplan Meyer plots for PFS and OS for different tumor locations and sex. (G) Overall survival (OS) remained unchanged between patients with or without distant progression (p = 0.9). (H) Spinal tumor location was not significantly associated with shorter PFS. 

*Supplementary Figure 3: Supplementary Figure 3. CNA in spinal versus non-spinal tumors and per patient:*

(A) Percentage of CNAs in cases with spinal tumors (red) and non-spinal tumors (green). No significant differences between CNA profiles were observed. (B) Percentage of the genome affected by CNAs per patient, including both gains and losses.

*Supplementary Figure 4. Flowchart of performed analyses:*

The numbers within each branch indicate the patients included in the respective analyses and their overlap.

Supplementary table 1: Patient characteristics of adult patient cohort

| **Age at diagnosis (years)** |  |
| --- | --- |
| Median ±  Standard deviation (range) | 31.8 ± 13.3 (18 – 77) |
| **Distant progression (percent)** |  |
| yes | 10 (19.2) |
| no | 42 (80.8) |
| **Sex (percent)** |  |
| Female | 22 (42.3) |
| Male | 30 (57.7) |
| **Location (percent)** |  |
| Thalamus | 19 (36.5) |
| Pons | 12 (23.1) |
| Spinal cord | 9 (17.3) |
| Cerebellum | 6 (11.5) |
| Cerebral hemispheres | 4 (7.7) |
| Medulla oblongata | 2 (3.8) |
| **Procedure to establish diagnosis (percent)** |  |
| Complete resection | 5 (9.6) |
| Partial resection | 19 (36.5) |
| Stereotactic biopsy | 28 (53.8) |
| **Adjuvant therapy (percent)** |  |
| Radiotherapy and Temozolomide | 29 (55.8) |
| Radiotherapy alone | 15 (28.8) |
| None | 8 (15.3) |
| **MGMT Promotor Hypermethylation (percent)** |  |
| Methylated | 5 (9.6) |
| Non-methylated | 27 (51.9) |
| Unknown | 20 (38.5) |

Supplementary table 2: Fusions and genes analyzed in panel sequencing

| **Genes** | **Fusions** | **Promoter** |
| --- | --- | --- |
| ABL1 | KIAA1549-FUSION | Tert-Promotor |
| ACVR1 | BRAF-FUSION |  |
| AIFM3 | C11ORF95-FUSION |  |
| AIP | FGFR1-FUSION |  |
| AKT1 | NAB2-FUSION |  |
| AKT2 | STAT6-FUSION |  |
| AKT3 | NTRK2-FUSION |  |
| ALK | ALK-FUSION |  |
| APC | ROS1-FUSION |  |
| ARID1A |  |  |
| ARID1B |  |  |
| ARID2 |  |  |
| ASXL1 |  |  |
| ATM |  |  |
| ATR |  |  |
| ATRX |  |  |
| ATXN1 |  |  |
| ATXN1L |  |  |
| BAP1 |  |  |
| BCOR |  |  |
| BRAF |  |  |
| BRCA1 |  |  |
| BRCA2 |  |  |
| BRPF1 |  |  |
| BRPF3 |  |  |
| C11ORF95 |  |  |
| CCND1 |  |  |
| CCND2 |  |  |
| CDH1 |  |  |
| CDK4 |  |  |
| CDK6 |  |  |
| CDKN2A |  |  |
| CDKN2C |  |  |
| CHD7 |  |  |
| CHEK2 |  |  |
| CIC |  |  |
| COL2A1 |  |  |
| CREBBP |  |  |
| CSF1R |  |  |
| CTDNEP1 |  |  |
| CTNNB1 |  |  |
| D2HGDH |  |  |
| DAXX |  |  |
| DDR1 |  |  |
| DDX3X |  |  |
| DGCR8 |  |  |
| DICER1 |  |  |
| DROSHA |  |  |
| EED |  |  |
| EGFR |  |  |
| EIF1AX |  |  |
| EPAS1 |  |  |
| ERBB2 |  |  |
| ESR1 |  |  |
| EZH2 |  |  |
| EZHIP |  |  |
| FBXW7 |  |  |
| FGFR1 |  |  |
| FGFR2 |  |  |
| FGFR3 |  |  |
| FGFR4 |  |  |
| FLT3 |  |  |
| FOXO3 |  |  |
| FOXR2 |  |  |
| FUBP1 |  |  |
| GABRA6 |  |  |
| GNA11 |  |  |
| GNAQ |  |  |
| GNAS |  |  |
| GPR161 |  |  |
| GSE1 |  |  |
| H2AFX |  |  |
| H2AX |  |  |
| H3C2 |  |  |
| H3C3 |  |  |
| H3F3A |  |  |
| H3F3B |  |  |
| H3P6 |  |  |
| HDAC2 |  |  |
| HIST1H3B |  |  |
| HIST1H3C |  |  |
| HNF1A |  |  |
| HRAS |  |  |
| HTRA1 |  |  |
| IDH1 |  |  |
| IDH2 |  |  |
| IDO2 |  |  |
| IKBKAP (ELP1) |  |  |
| IST1 |  |  |
| JAK2 |  |  |
| JAK3 |  |  |
| KBTBD4 |  |  |
| KCNK12 |  |  |
| KDM6A |  |  |
| KDR |  |  |
| KIT |  |  |
| KLF4 |  |  |
| KLK1 |  |  |
| KMT2B |  |  |
| KMT2C |  |  |
| KMT2D |  |  |
| KRAS |  |  |
| LDB1 |  |  |
| LRRFIP2 |  |  |
| LZTR1 |  |  |
| MDM2 |  |  |
| MDM4 |  |  |
| MEN1 |  |  |
| MET |  |  |
| MGMT |  |  |
| MLH1 |  |  |
| MLL2 |  |  |
| MN1 |  |  |
| MPL |  |  |
| MRE11 |  |  |
| MRE11A |  |  |
| MSH2 |  |  |
| MSH6 |  |  |
| MTOR |  |  |
| MYB |  |  |
| MYBL1 |  |  |
| MYC |  |  |
| MYCN |  |  |
| MYCNOS |  |  |
| MYL1 |  |  |
| NAB2 |  |  |
| NBN |  |  |
| NDRG2 |  |  |
| NDUFS3 |  |  |
| NF1 |  |  |
| NF2 |  |  |
| NOTCH1 |  |  |
| NRAS |  |  |
| NTRK2 |  |  |
| PALB2 |  |  |
| PAX6 |  |  |
| PBRM1 |  |  |
| PCDH8 |  |  |
| PDGFRA |  |  |
| PHOX2B |  |  |
| PIK3C2B |  |  |
| PIK3C2G |  |  |
| PIK3CA |  |  |
| PIK3CG |  |  |
| PIK3R1 |  |  |
| PIK3R2 |  |  |
| PLCH1 |  |  |
| PLXNB1 |  |  |
| PMS2 |  |  |
| POLD1 |  |  |
| POLE |  |  |
| PPL |  |  |
| PPM1D |  |  |
| PRKAR1A |  |  |
| PRKCA |  |  |
| PTCH1 |  |  |
| PTCH2 |  |  |
| PTEN |  |  |
| PTPMT1 |  |  |
| PTPN11 |  |  |
| RAD50 |  |  |
| RAF1 |  |  |
| RB1 |  |  |
| RBPJ |  |  |
| RELA |  |  |
| RET |  |  |
| ROS1 |  |  |
| SDCCAG8 |  |  |
| SETD2 |  |  |
| SF3B1 |  |  |
| SH3PXD2A |  |  |
| SMAD4 |  |  |
| SMARCA2 |  |  |
| SMARCA4 |  |  |
| SMARCB1 |  |  |
| SMARCD1 |  |  |
| SMARCD2 |  |  |
| SMARCD3 |  |  |
| SMARCE1 |  |  |
| SMIM4 |  |  |
| SMO |  |  |
| SPRED1 |  |  |
| SRP19 |  |  |
| STAG2 |  |  |
| STAT6 |  |  |
| SUFU |  |  |
| SUZ12 |  |  |
| TBR1 |  |  |
| TCF4 |  |  |
| TERT |  |  |
| TH2LCRR |  |  |
| TP53 |  |  |
| TRAF7 |  |  |
| TSC1 |  |  |
| TSC2 |  |  |
| TSPAN31 |  |  |
| VHL |  |  |
| YAP1 |  |  |
| ZIC1 |  |  |
| ZIC4 |  |  |
| ZMYM3 |  |  |

Supplementary table 3: Most common CNV events, stratified by distant progression vs. no progression and gains or losses of whole chromosomal arms

| Distant progression | CNV | Chromosome | N samples with CNV | n_total in gorup | percent |
| --- | --- | --- | --- | --- | --- |
| no | gain | chr21 | 11 | 26 | 42.3 |
| no | gain | chr12 | 10 | 26 | 38.5 |
| no | gain | chr22q | 10 | 26 | 38.5 |
| no | gain | chr17 | 8 | 26 | 30.8 |
| no | gain | chr18 | 7 | 26 | 26.9 |
| no | gain | chr1 | 6 | 26 | 23.1 |
| no | gain | chr13q | 6 | 26 | 23.1 |
| no | gain | chr14q | 6 | 26 | 23.1 |
| no | gain | chr15q | 6 | 26 | 23.1 |
| no | gain | chr16 | 6 | 26 | 23.1 |
| no | loss | chr5 | 16 | 26 | 61.5 |
| no | loss | chr11 | 15 | 26 | 57.7 |
| no | loss | chr14q | 12 | 26 | 46.2 |
| no | loss | chr6 | 12 | 26 | 46.2 |
| no | loss | chr15q | 11 | 26 | 42.3 |
| no | loss | chr13q | 9 | 26 | 34.6 |
| no | loss | chr8 | 9 | 26 | 34.6 |
| no | loss | chr10q | 8 | 26 | 30.8 |
| no | loss | chr20 | 8 | 26 | 30.8 |
| no | loss | chr3 | 8 | 26 | 30.8 |
| yes | gain | chr9 | 4 | 7 | 57.1 |
| yes | gain | chr20 | 3 | 7 | 42.9 |
| yes | gain | chr22q | 3 | 7 | 42.9 |
| yes | gain | chr15q | 2 | 7 | 28.6 |
| yes | gain | chr17 | 2 | 7 | 28.6 |
| yes | gain | chr18 | 2 | 7 | 28.6 |
| yes | gain | chr19 | 2 | 7 | 28.6 |
| yes | gain | chr3 | 2 | 7 | 28.6 |
| yes | gain | chr10p | 1 | 7 | 14.3 |
| yes | gain | chr11 | 1 | 7 | 14.3 |
| yes | loss | chr5 | 5 | 7 | 71.4 |
| yes | loss | chr14q | 4 | 7 | 57.14 |
| yes | loss | chr3 | 4 | 7 | 57.1 |
| yes | loss | chr6 | 4 | 7 | 57.1 |
| yes | loss | chr9 | 4 | 7 | 57.1 |
| yes | loss | chr10 | 3 | 7 | 42.9 |
| yes | loss | chr8 | 3 | 7 | 42.9 |
| yes | loss | chr1 | 2 | 7 | 28.6 |
| yes | loss | chr13q | 2 | 7 | 28.6 |
| yes | loss | chr15q | 2 | 7 | 28.6 |

Supplementary table 4: Most common CNV events, stratified by spinal location vs. non spinal location and gains or losses of whole chromosomal arms

| spinal | CNV | unit | n_samples | n_total | percent |
| --- | --- | --- | --- | --- | --- |
| no | gain | chr21 | 10 | 26 | 38.5 |
| no | gain | chr22q | 10 | 26 | 38.5 |
| no | gain | chr12 | 9 | 26 | 34.6 |
| no | gain | chr17 | 7 | 26 | 26.9 |
| no | gain | chr1 | 6 | 26 | 23.1 |
| no | gain | chr18 | 6 | 26 | 23.1 |
| no | gain | chr13q | 5 | 26 | 19.2 |
| no | gain | chr14q | 5 | 26 | 19.2 |
| no | gain | chr15q | 5 | 26 | 19.2 |
| no | gain | chr16 | 5 | 26 | 19.2 |
| no | loss | chr11 | 16 | 26 | 61.5 |
| no | loss | chr5 | 16 | 26 | 61.5 |
| no | loss | chr14q | 13 | 26 | 50.0 |
| no | loss | chr6 | 13 | 26 | 50.0 |
| no | loss | chr15q | 12 | 26 | 46.2 |
| no | loss | chr8 | 10 | 26 | 38.5 |
| no | loss | chr10q | 8 | 26 | 30.8 |
| no | loss | chr13q | 8 | 26 | 30.8 |
| no | loss | chr19q | 8 | 26 | 30.8 |
| no | loss | chr3 | 8 | 26 | 30.8 |
| yes | gain | chr20 | 4 | 7 | 57.1 |
| yes | gain | chr9 | 4 | 7 | 57.1 |
| yes | gain | chr15q | 3 | 7 | 42.9 |
| yes | gain | chr17 | 3 | 7 | 42.9 |
| yes | gain | chr18 | 3 | 7 | 42.9 |
| yes | gain | chr19 | 3 | 7 | 42.9 |
| yes | gain | chr22q | 3 | 7 | 42.9 |
| yes | gain | chr12 | 2 | 7 | 28.6 |
| yes | gain | chr14q | 2 | 7 | 28.6 |
| yes | gain | chr10p | 1 | 7 | 14.3 |
| yes | loss | chr5 | 5 | 7 | 71.4 |
| yes | loss | chr3 | 4 | 7 | 57.1 |
| yes | loss | chr13q | 3 | 7 | 42.9 |
| yes | loss | chr14q | 3 | 7 | 42.9 |
| yes | loss | chr18 | 3 | 7 | 42.9 |
| yes | loss | chr6 | 3 | 7 | 42.9 |
| yes | loss | chr9 | 3 | 7 | 42.9 |
| yes | loss | chr1 | 2 | 7 | 28.6 |
| yes | loss | chr10 | 2 | 7 | 28.6 |
| yes | loss | chr16 | 2 | 7 | 28.6 |
